# Store-operated Ca^2+^ entry supports contractile function in hearts of hibernators

**DOI:** 10.1371/journal.pone.0177469

**Published:** 2017-05-22

**Authors:** Olga V. Nakipova, Alexey S. Averin, Edward V. Evdokimovskii, Oleg Yu. Pimenov, Leonid Kosarski, Dmitriy Ignat’ev, Andrey Anufriev, Yuri M. Kokoz, Santiago Reyes, Andre Terzic, Alexey E. Alekseev

**Affiliations:** 1 Institute of Cell Biophysics, Russian Academy of Sciences, Pushchino, Moscow Region, Russia; 2 Institute of Theoretical and Experimental Biophysics, Russian Academy of Science, Pushchino, Moscow Region, Russia; 3 Institute of Biology, Yakutsk Branch, Siberian Division, Russian Academy of Sciences, Yakutsk, Russia; 4 Division of Cardiovascular Diseases, Department of Molecular Pharmacology and Experimental Therapeutics, Stabile 5, Mayo Clinic, Rochester, Minnesota, United States of America; University of Debrecen, HUNGARY

## Abstract

Hibernators have a distinctive ability to adapt to seasonal changes of body temperature in a range between 37°C and near freezing, exhibiting, among other features, a unique reversibility of cardiac contractility. The adaptation of myocardial contractility in hibernation state relies on alterations of excitation contraction coupling, which becomes less-dependent from extracellular Ca^2+^ entry and is predominantly controlled by Ca^2+^ release from sarcoplasmic reticulum, replenished by the Ca^2+^-ATPase (SERCA). We found that the specific SERCA inhibitor cyclopiazonic acid (CPA), in contrast to its effect in papillary muscles (PM) from rat hearts, did not reduce but rather potentiated contractility of PM from hibernating ground squirrels (GS). In GS ventricles we identified drastically elevated, compared to rats, expression of Orai1, Stim1 and Trpc1/3/4/5/6/7 mRNAs, putative components of store operated Ca^2+^ channels (SOC). Trpc3 protein levels were found increased in winter compared to summer GS, yet levels of Trpc5, Trpc6 or Trpc7 remained unchanged. Under suppressed voltage-dependent K^+^, Na^+^ and Ca^2+^ currents, the SOC inhibitor 2-aminoethyl diphenylborinate (2-APB) diminished whole-cell membrane currents in isolated cardiomyocytes from hibernating GS, but not from rats. During cooling-reheating cycles (30°C–7°C–30°C) of ground squirrel PM, 2-APB did not affect typical CPA-sensitive elevation of contractile force at low temperatures, but precluded the contractility at 30°C before and after the cooling. Wash-out of 2-APB reversed PM contractility to control values. Thus, we suggest that SOC play a pivotal role in governing the ability of hibernator hearts to maintain their function during the transition in and out of hibernating states.

## Introduction

Adequate cardiac function relies on versatile regulation of intracellular Ca^2+^ levels ([Ca^2+^]_i_) to ensure coordination of multiple Ca^2+^-dependent processes in response to various environmental factors [1]. Indeed, dysregulation of such high fidelity mechanism in cardiac cells under conditions of stress, food restriction, hypoxia, changes in body temperature, etc. may induce arrhythmia and ventricular fibrillation, myocardial ultrastructural damages along with necrosis or apoptosis [2–5], which all can be life threatening. Survivorship under severe environmental pressures evolutionarily fixed the superior regulation of intracellular Ca^2+^ concentrations in hibernating animals compared to non-hibernating species. Therefore, an assessment of the Ca^2+^ regulation plasticity in hibernators would be tantamount not only to understanding their surviving mechanisms but also to identifying more effective therapeutic strategies against cardiac diseases [6,7].

During winter, hibernating animals are capable of enduring a deep hypothermia with an extremely diminished metabolic activity and recover to normal levels after arousal [8–10]. At a body temperature close to 0°C, hibernators survive through prolonged periods of lowered hemodynamics, and following rewarming can restore normal circulation without detrimental consequences for the whole organism [11–13]. Heart rates of a prototypic hibernating species, the ground squirrel (*Spermophilus undulatus*), increase during arousal from 2–3 to 350–600 beats/min along with rising thermogenesis and decrease to 130–160 beats/min at the normal active state [14–16]. Thus, during hibernation—arousal transitions, hibernators have to outlive a set of cardiovascular traits that would be fatal to humans and other non-hibernating mammals, e.g. violent swings in body temperature [8,17], extreme sympathetic drive during arousal [18–20], blood viscosity [21], oxidative stress [22,23], lethal ventricular fibrillation and cardiac arrhytmias [24], etc. While such deleterious conditions in cells from non-hibernating animals would normally deregulate the control of Ca^2+^-dependent processes, cardiomyocytes from mammalian hibernators exhibit a remarkable ability to adapt intracellular Ca^2+^ maintenance and, thereby, contractile function [6,25,26]. Indeed, in contrast to cardiomyocytes from non-hibernators that at 5–10°C are characterized by a more than two-fold increase of resting [Ca^2+^]_i_ and reduced contractile function, ventricular myocytes from ground squirrels at low temperatures are capable of supporting nominal resting [Ca^2+^]_i_ [6]. It has been established that the rate of excitation-induced [Ca^2+^]_i_-transient decays, as a measure of SERCA activity, was more resistant to temperature drops in ground squirrel compared to rat cardiac muscles [25,27]. Moreover, hibernating ground squirrels apparently are capable of increasing their capacity to accumulate Ca^2+^ in SR compared to non-hibernating animals [28], which may be a reason for the elevated excitation-induced amplitude of [Ca^2+^]_i_ dynamics [6,7]. This paradoxical increase in cytosolic Ca^2+^ load appears to be critical to retain forceful contraction at the decreased Ca^2+^ sensitivity of myofilaments identified at low temperatures in hibernator myofibrils [29,30]. Actually, at low temperatures, cardiac muscles from hibernating ground squirrels are characterized by even higher contraction force compared to contractility measured at normal body temperatures [27,31].

During hibernation, influx of extracellular Ca^2+^ to cardiomyocytes is significantly reduced due to downregulation of voltage-gated L-type Ca^2+^ channels and suppression of sympathetic tone [19,32] that would otherwise maintain Ca^2+^ channels operative via PKA-dependent phosphorylation linked to the targeting of β-adrenoceptors by catecholamines [1]. While involvement of L-type Ca^2+^ current into excitation-contraction coupling in hibernators cannot be completely ruled out, the main contributors to [Ca^2+^]_i_, necessary for myocardial contraction, are deemed to be Ca^2+^ influx through the Na^+^-Ca^2+^ exchanger (NCX) and intracellular Ca^2+^ release mechanisms [33,34]. However, it remains unclear why, at low temperatures, enzymatic reactions with higher Arrhenius activation energy compared to ion diffusion via channels down its electrochemical gradient would preponderate in cells of hibernators. It is possible that near the freezing point the voltage-sensing conformational rearrangements within the Ca^2+^ channel complex may become malfunctioning to drive openings of remaining operational Ca^2+^ channels. If so, voltage-gated L-type Ca^2+^ channels, although potentially can provide a passive Ca^2+^ ion influx, may not be a reliable Ca^2+^ transporting system, for example during the transition from deep torpor to arousal when the demand for increased circulation precedes rewarming of cardiac tissue [19].

Alternatively, hibernators may evolutionary conserve another mechanism capable of increasing cytoplasmic [Ca^2+^]_i_ that phylogenetically is more basic than ion transport via voltage-gated Ca^2+^ channels or NCX, namely, the store-operated Ca^2+^ channel entry (SOCE). Indeed, in contrast to voltage-gated Ca^2+^ channels that are typical for excitable tissues, SOCE are widely spread in all eukaryotes from yeast to humans and, thereby, represent the primordial Ca^2+^ entry pathway [35]. Discovery of the stromal interaction molecule 1 (STIM1) as an ER Ca^2+^ sensor, and Orai1 as a functional membrane component of store-operated channels (SOC), was a key step in defining the mechanism of SOCE [36,37]. Additional functional units for SOC, albeit still under debate, are the transient receptor potential (TRP) channels [38–41]. In particular, the canonical TRP family (TRPC), which includes 7 isoforms of nonselective cation channels (TRPC1-7), has been identified in cardyomyocytes under pathological conditions [42–45]. However, the expression and activity of SOC in ventricular cardiac muscles of hibernating mammals are unknown. Herein, we assess the contribution of SOC in contractile adaptation of papillary muscle from hibernating ground squirrels, compared to non-hibernating rats, under conditions involving depletion/replenishment of intracellular Ca^2+^ stores and within the temperature range characterizing transitions between torpid and active states.

## Materials and methods

### Experimental animals and ethics statement

This study did not involve endangered or protected species and all animal procedures performed with male Sprague-Dawley rats (obtained from laboratory of experimental animals, Pushchino, Russia) and ground squirrels, *Spermophilus undulatus*, were approved by the Biological Safety and Ethics Committee (Institute of Cellular Biophysics) in accord with Directive 2010/63/EU of the European Parliament. Ground squirrels were live trapped during August—September in Yakutia, where this species is abandoned and unprotected, by trained personnel of the laboratory of Ecology and Ecosystem Resilience of North (Institute of Biology, Yakutsk Branch of Russian Academy of Sciences). Animals were shipped by air to Pushchino, the Moscow region, housed in a specially equipped vivarium and fed *ad libitum*. In October, prior to the beginning of endogenous hibernating cycle. the temperature in the vivarium was kept at ~4°C with no light to prepare animals for hibernation. This study implemented two experimental groups of active ground squirrels: 1) active winter animals (January–the first half of March) included animals taken between bouts of hibernation (interbout), during 12–24 h after spontaneous arousing; 2) summer group (June–July) included animals that were removed from the cold room in April and kept at room temperature. The averaged rectal temperature of animals in the both experimental groups was 36.5 ± 0.5°C. All surgery was performed under sodium pentobarbital anesthesia (50 mg/kg i.p.), and all efforts were made to minimize suffering.

### Contractility of papillary muscles

Isolations of right ventricle papillary muscles were performed from hearts of anesthetized animals. Measurements of the isometric force of contraction of rat and ground squirrel papillary muscles were performed in oxygenated (95% O_2_/5% CO_2_) Tyrode solution containing (in mM): NaCl, 135; KCl, 4; MgCl_2_, 1; CaCl_2_, 1.8; NaHCO_3_, 13.2; KH_2_PO_4_, 1.8; glucose, 11; (pH 7.4) as previously described [46]. In brief, isolated papillary muscles (length 2–3 mm, cross-sectional area 0.45 ± 0.07 mm^2^) were mounted horizontally in a temperature controlled chamber (30°C), and stretched to a length at which tension of contraction is maximal. Isometric force of contraction was measured using a SI-H KG series force transducer (WPI Germany GmbH). Stimuli were applied using bipolar Ag-AgCl electrodes by square-wave pulses of 5 ms duration and amplitude set at 25% above the excitation threshold. Prior to each experiment, muscle preparations were stimulated at 0.3 Hz for 1 h until complete mechanical stabilization. All experiments were performed using a customized heating/cooling temperature controller engineered based on a Peltier thermocouple with feedback control to the targeted temperature.

### RNA isolation and RT-qPCR

Total RNA was extracted from papillary muscles of rats and winter active (interbout) ground squirrels using ExtractRNA reagent (Evrogen, Moscow, Russia). Treatment of RNA samples with DNAase was followed by chloroform extraction and subsequent precipitation in 96% ethanol. RNA pellets were washed three times with 70% ethanol and dissolved in RNase-free water. Synthesis of cDNA was carried out with the commercially available reverse transcription MMLV RT kit (Evrogen, Moscow, Russia) using an oliogo(dT)_18_ primer. qPCR for *Cav1*.*2*, *Orai1*, *Stim1* and *Trpc* isoforms was performed with the ABI 7500 Real-Time System (Thermo Fisher Scientific Inc., MA, USA) using a qPCRmix-HS SYBR+LowRox kit (Evrogen) with the forward (F) and reverse (R) primers indicated in Table 1. At least one primer in each pair corresponds to an exon junction in all genes studied. The amplification efficiency for all implemented genes were experimentally confirmed to be 90–105% based on a standard calibration procedure by titrating corresponding cDNA samples from the species compared. The sizes of all amplicons were close to each other and not exceeding 70–100 base pairs. The reaction was initially incubated at 95°C for 20 s and then for 40 cycles consisting of denaturation at 95°C for 20 s and annealing/extension at 60°C for 40 s. The threshold cycle (C_T_) was determined by the 7000 System SDS software (ver. 1.3.1; Applied Biosystems). Transcript levels were quantified by the 2^-ΔΔCT^ method taking into consideration the identified amplification efficiencies implementing REST 2005 software (Corbett Life Science, Munich, Germany) specialized for these purposes [47,48].

**Table 1:** Primer sequences for PCR amplification.

	RAT	GROUND SQUIRREL
*Stim1*	(F) GTCGCCCTTGTCCATGCAG (R) ATGGGTCAAATCCCTCTGAGATCC	(F) CAGTTCTCATGGCCCGAGTT (R) GTGGGGAATGCGTGTGTTTC
*Orai1*	(F) CGCAAGCTCTACTTGAGCCG (R) CATCGCTACCATGGCGAAGC	(F) GCATCGCCACATCGAGCTA (R) AGAACTTGACCCAGCAGAGC
*Trpc1*	(F) GCAGAACAGCTTGAAGGAGTG (R) CACTAGGCAGCACATCACCT	(F) ACAGATCAGGCAACTGTGGAA (R) GAAGTCCGAAAGCCAAGCAA
*Trpc3*	(F) AGGTGAACGAAGGTGAACTGA (R) TCCGTCGCTTGGCTCTTATC	(F) GCCTTCGGTATGAGCTTTTGG (R) GCTTCTCGCTGAGTTTGTGG
*Trpc4*	(F) GGTTGTCCTCCTGAACATGC (R) ATATCTGCGTGGTCGGCAAT	(F) AGCAGATTCAGACGAAAAGAGTG (R) TGCTGCTGACCTTGGATGAA
*Trpc5*	(F) TCTGTCCCAAGAGAGACCCC (R) CAGCATGACGTTCTGTGAAGC	(F) GCTTTTCCACAAGCAGCACT (R) GAGACGCTCTTGGATTTGGC
*Trpc6*	(F) AAAGATACGTACTGCAGGCCC (R) ATTTCCTTCAGCTCCCCTTCG	(F) TCAAGTCTCCGTTATGAACTCCT (R) CCTCTTGATTTGGCTCCAAGG
*Trpc7*	(F) AGGCCAAACGCTGTGAAAAC (R) CCTGGTAGCGAGTCTTCCTG	(F) GAAGTCCCAAGCTACTGGCG (R) CACCCTCAGGTGGTCTTTGTT
*Gapdh*	(F) TCTCTGCTCCTCCCTGTTCTA (R) GCCAAATCCGTTCACACCG	(F) CAACGCTGGCATATCCCTCA (R) CCACCACCCGATGACTGTAG
*Cav1*.*2*	(F) GGGCAGTTTGCTCAAGATCC (R) CGCGTTCTCCATCTCCTCTAT	

### Immunoblotting

Isolated papillary muscles from interbout and summer ground squirrels were lysed in hypotonic buffer containing NaCl 20 mM, Tris-HCl 20 mM (pH 7.4) and 1% Triton X-100, supplemented with proteinase inhibitors and centrifuged at 2,500∙*g* for 5 minutes. Proteins were separated by denaturing 10% polyacrylamide gel electrophoresis (SDS-PAGE) and transferred to a nitrocellulose membrane (0.45 μm; Santa-Cruz, sc-3724). Primary rabbit polyclonal antibodies (Abcam) against Trpc3 (ab51560) and Trpc5 (ab63151), mouse polyclonal antibodies against Trpc6 (ab63038) and Trpc7 (ab93618) and goat primary antibody against actin (Santa Cruz, sc-1616) were diluted at 1:1000 and used to probe immunoreactive proteins. Counterstain was performed with horseradish peroxidase (HRP)-conjugated anti-rabbit (Santa Cruz, sc-2004, 1:300 dilution), anti-mouse (Abcam, ab131368, 1:300 dilution) or anti-goat (Santa Cruz, sc-2020) secondary antibodies, respectively. HRP signals were detected using 3,3'-diaminobenzidine tetrahydrochloride (DAB) substrate (Amresco, E733) and film-captured.

### Isolation of cardiac myocytes and patch-clamp measurements

Hearts were dissected from anesthetized animals, and retrogradely perfused with “low-Ca^2+^ medium” containing (in mM): NaCl, 80; KCl, 10; KH_2_PO_4_, 1.2; MgSO_4_, 5; glucose, 20; taurine, 50; L-arginine, 1; HEPES, 10 (pH 7.2), as described previously [32]. Cardiomyocytes were isolated as described [32] and stored in low-Ca^2+^ medium supplemented with 200 μM CaCl_2_. Only rod-shaped cardiomyocytes with clear striations were used. Membrane currents in isolated cardiac myocytes were measured using the perforated mode of the whole-cell patch clamp technique, as described [32]. Membrane patch perforation was induced by amphotericin B (200–250 μg/mL) added to the pipette (4–5 MΩ) containing (in mM): CsCl, 130; MgCl, 5; HEPES, 10 (pH 7.25). The bath solution contained (in mM): Choline-Cl, 80; CsCl, 10; MgCl_2_, 2; CaCl_2_, 1.8; TEA-Cl, 20; Glucose 1g/L; TRIS 10, pH 7.25 with HCl. In pipette and bath solutions cesium and TEA ions were used to diminish K^+^ currents. L-type Ca^2+^ currents were suppressed by 10 μM of nifedipine. Currents were measured using an Axopatch 200B amplifier (Molecular Devices, USA). Cellular membrane resistance along with cell capacitance were defined online based on analysis of capacitive transient currents using the custom BioQuest software [32] and a L-154 AD/DA converter (L-card, Moscow, Russia). Series resistance, compensated by 50–60%, and uncompensated cell capacitances were continuously monitored for the quality of the whole-cell recording configuration throughout experiments. Measurements were performed at 20–22°C.

### Data analysis and statistics

Student’s t-test was used to compare continuous variables. P value of 0.05 was predetermined as determining statistically significant differences. All data are presented as mean ± standard error (S.E.). Results from RT-qPCR were presented using Whisker-Box plots, where the box area encompasses 50% of all observations, the dotted line represents the sample median and the whiskers shows 95% confidential intervals.

## Results

### Contractility of papillary muscles of ground squirrels versus rats during cooling-reheating cycle

**Fig 1:**
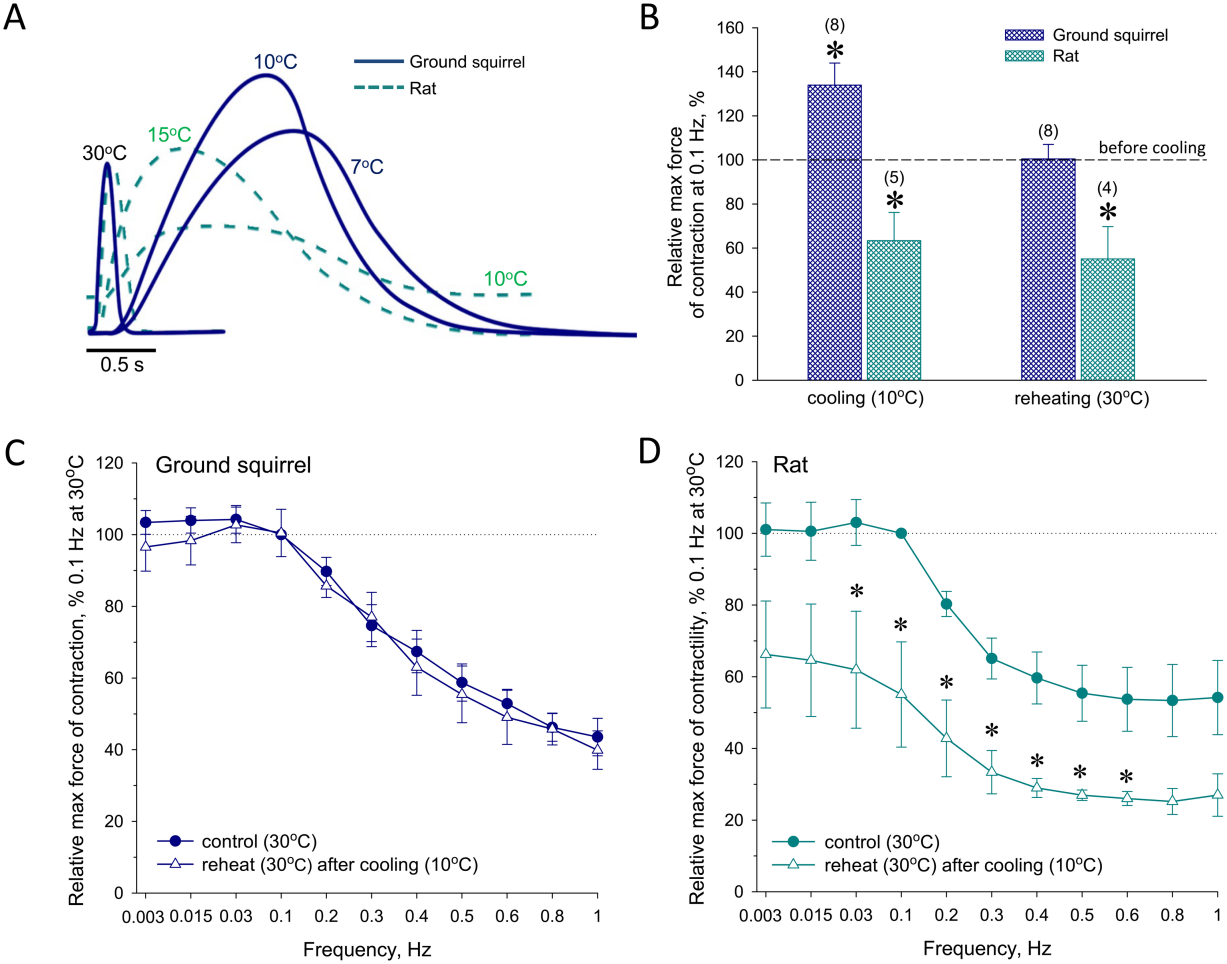
Temperature-dependent adaptation of papillary muscle contractility in ground squirrel and rat hearts. (**A**) representative relative isometric contractile forces measured at 0.1 Hz stimulation frequency in isolated papillary muscles from ground squirrel (solid lines) and rat (dotted lines) hearts at different temperatures (as indicated). Contractile forces in both species were plotted relative to the values obtained at 30°C. (**B**) Summary statistics for the contractility of papillary muscle under conditions indicated in (**A**); *, denotes statistically significant differences (P<0.05, n shown in parenthesis) estimated using the single group t-test compared to maximal force of contraction before cooling (100%). (**C** and **D**) FFR constructed at 30°C before cooling to 10°C and after reheating; point values were obtained by normalizing measured maximal contraction forces to the averaged value at 0.1 Hz before cooling; *, denotes statistically significant differences between values before cooling and after reheating (P<0.05; n = 7 in ground squirrels, and n = 4 in rats).

Initial contractions at 30°C in both cardiac papillary muscles (PM) from interbout ground squirrels and rats were designated as zero, baseline values, and isometric contraction forces were measured at reduced ambient temperatures in response to 0.1 Hz stimulus (Fig 1A). During cooling, PM of winter interbout ground squirrel exhibited significantly increased contractility, which remained above control values until reaching 7°C (Fig 1A and 1B). PM of rat hearts were also characterized by potentiation of peak developed forces during cooling until 15°C, which was however followed by gradual elevation of diastolic tension and significant reduction of peak contractile values upon continuation of the cooling process (Fig 1A and 1B). Remarkably, PM contractility returned to control values after rewarming back to 30°C in ground squirrels, but not in rats (Fig 1B), emphasizing a superior adaptive plasticity of cardiac muscle function in hibernators. Of note, PM of summer ground squirrel, although did not demonstrate the substantial potentiation of contractility during cooling, were capable of fully restoring contractile function after reheating in contrast to rats (S1 Fig). This unique feature of hibernators’ hearts was further confirmed using the contractile force-frequency relationship (FFR), an important characteristic of the cardiac contractile reserve, which allows discriminating between diverse calcium-transporting systems contributing to the control of contraction force [49,50]. It has been established that, at low frequencies, the main mediator of contractile function is intracellular Ca^2+^ release from sarcoplasmic reticulum (SR), supported by SERCA, whereas at high stimulation frequencies the force of contraction is primarily controlled by Ca^2+^ influx via L-type Ca^2+^ channels [46,51–53]. Within the frequency range from 0.003 to 1 Hz, the constructed FFR displayed a negative staircase response (decreased force with increasing frequency), typical for mature rats as well as for hibernating and interbout ground squirrels [46,49,51]. The FFR exhibited complete reversibility of contractile function upon rewarming within the whole frequency range in PM of ground squirrels, but not in rats (Fig 1C and 1D).

### Suppression of SERCA induced opposite effects on PM contractility in ground squirrels and rats

**Fig 2:**
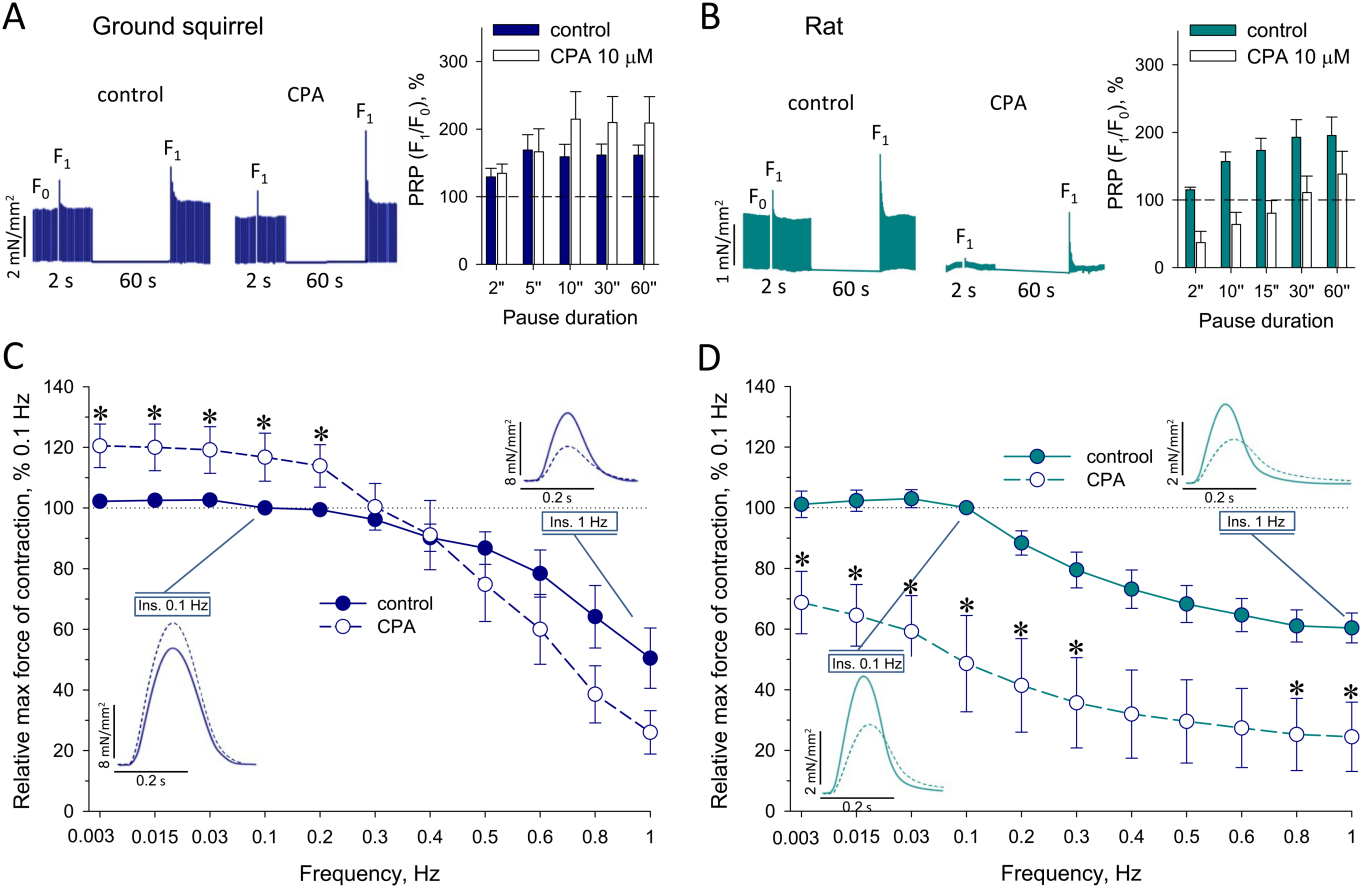
CPA-induced changes in PRP and FFR of PM contractile function. (**A** and **B**) Representative recordings of ground squirrel and rat PM contractility implementing 2 s- and 60 s-long pauses along with corresponding PRP, calculated as a percentile ratio of the first post-rest contraction force **F**_**1**_ to the basal rhythmic contraction force **F**_**0**_ (at 1 Hz), plotted as a function of pause durations in the absence and presence of CPA. (**C**) and (**D**) Ground squirrels and rat FFR in the absence and presence of 10 μM CPA were constructed as described in Fig 1C and 1D. Insets at 0.1 and 1 Hz represent typical changes in PM contractility induced by CPA (dotted lines); *, denotes statistically significant CPA-induced changes in relative force contraction values (P<0.05; n = 9 in ground squirrels, and n = 3 in rats).

It has been shown that post rest potentiation (PRP) of PM contractility can serve as a qualitative parameter of SR Ca^2+^ levels, and normally should correlate well with contractile pause durations, during which SR Ca^2+^stores can be refilled by SERCA [11,54,55]. Therefore, the observed weak negative FFR may be explained by abundant Ca^2+^ levels within SR, which, in turn, may be a consequence of relatively high spatial Na^+^ accumulation limiting removal of intracellular Ca^2+^ via reverse mode of NCX operation [46,50]. Indeed, the PRP in ground squirrel PM were marginal and statistically independent from the pause durations (Spearman’s rank correlation coefficient *ρ* = 0.3, P = 0.68; Fig 2A), whereas the PRP in rat PM correlated well with this parameter (*ρ* = 1.0, P = 0.017; Fig 2B). Suppression of SERCA activity by cyclopiazonic acid (CPA) considerably attenuated PM contractility in rats throughout the frequency stimulation range, which however retained the correlation of PRP with pause durations (Fig 2B and 2D). Conversely, in PM of interbout ground squirrels, but not in PM of summer animals, CPA paradoxically stimulated PRP, which remained independent from pause durations (*ρ* = 0.6, P = 0.35), and improved force of contraction at the stimulation frequencies below 0.3 Hz (Fig 2A and 2C and S2 Fig). Concomitantly, while CPA significantly delayed the contractile relaxation time TP_50_ in rat PM, such prolongations of TP_50_ in ground squirrel PM were statistically insignificant (Table 2). These data suggest that, in contrast to rats, ground squirrels can implement an alternative mechanism of intracellular Ca^2+^ handling capable of controlling myocardial contractility, such as store-operated Ca^2+^ entry.

**Table 2:** Changes of time-to-peak (TPT) and time to 50% relaxation (TP_50_) contractile parameters induced by CPA (10 μM) in isolated PM.

		n	TPT (ms)			TP_50_ (ms)		
			0.1 Hz	0.3 Hz	1 Hz	0.1 Hz	0.3 Hz	1 Hz
Ground squirrels	control	5	111.5±3.0	110.5±3.1	103.8±3.1	59.0±2.9	58.1±3.0	51.9±3.9
	CPA	5	123.7±4.5	118.5±1.9	108.9±3.6	69.7±5.0	65.9±6.4	62.6±7.5
Rat	control	4	113.4±2.4	109.8±2.9	104.7±2.8	60.0±2.3	63.2±1.2	59.6±1.4
	CPA	4	118.6±8.8	116.0±9.7	114.1±8.7	73.0±2.0*	80.4±1.6*	82.1±4.1*

*—significantly different (P<0.05) from corresponding controls.

### Comparative expression of SOC in papillary muscles of ground squirrels versus rats

**Fig 3:**
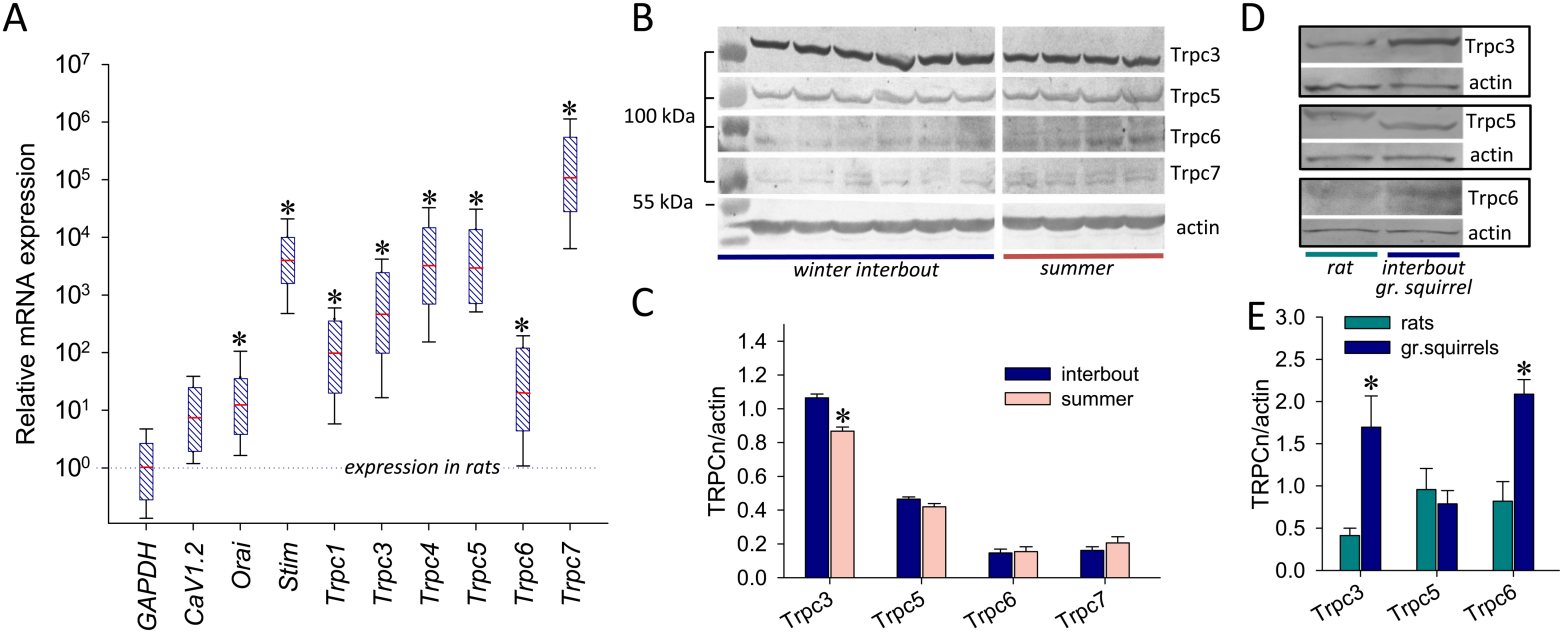
Expression of SOC components in papillary muscles (PM) from ground squirrel hearts. (**A**) Relative mRNA expression defined by qPCR in PM of ground squirrels. mRNA levels were normalized to housekeeping genes (GAPDH) and expressed as fold change of that determined in rat PM. *, denotes statistically significant difference with P<0.05 (n = 6–7 in both species). (**B**) and (**C**) Comparative Trpc3, Trpc5, Trpc6 and Trpc7 protein expression determined by western blot in PM from winter interbout (n = 6) versus summer ground squirrels (n = 4). (**D**) and (**E**) Comparative Trpc3, Trpc5 and Trpc6 protein expression determined by western blot in PM from winter interbout versus rats hearts (n = 3). Protein levels were expressed relative to β-actin; *, denote statistically significant expression difference with P < 0.05.

Comparative quantitative analysis of gene product expression using qPCR revealed substantially increased mRNA levels of putative myocardial SOC components in PM from hearts of interbout ground squirrels versus rat PM (Fig 3A). There was a >10^3^-fold increase in relative expression of *Stim1*, *Trpc4*, and *Trpc5*, as well as the 10^6^-fold increase in *Trpc7* mRNAs in ground squirrels compared to rat PM, indicating that SOCE may contribute to adaptive function of hibernating hearts. We did not find differences in the mRNA expression levels of all *Trpc*, *Stim1 and Orai1* genes in the summer versus winter ground squirrel groups. However, while there was no a significant difference in the expression of Trpc5, Trpc6 and Trpc7 proteins between active winter and summer ground squirrels, Trpc3, which exhibited significantly lower protein levels in summer compared to interbout ground squirrels (Fig 3B and 3C), may be a candidate participating in cardiac seasonal adaptation of hibernating ground squirrels. Of note, Trpc3 and Trpc6 proteins exhibited a marked increase of expression in hibernating ground squirrels compared to rats (Fig 3D and 3E).

### Ca^2+^ store-dependent regulation of papillary muscle contractility

**Fig 4:**
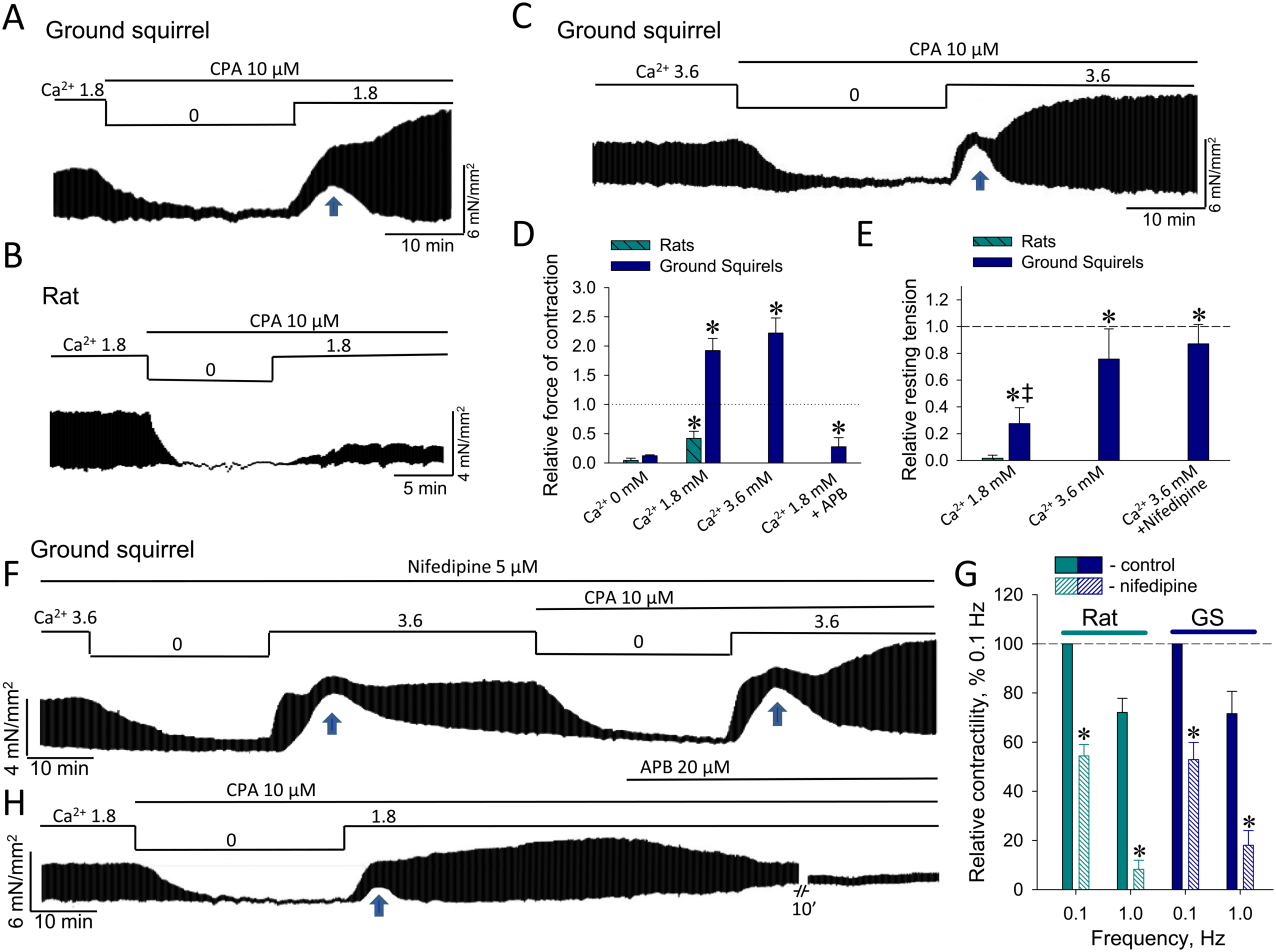
Ca^2+^ store-dependent regulation of contractile function of ground squirrel versus rat PM. (**A** and **B**) Representative recordings of ground squirrel and rat PM contractility under transition from nominal 1.8 mM Ca^2+^ to Ca^2+^-free Tyrode solution at 0.1 Hz stimulation frequency. (**C**) Same protocol in ground squirrel PM at doubled external Ca^2+^ levels also increase the force of contraction and further enhanced the amplitude of resting tension. Arrows in panels point to an increase in resting tension observed in ground squirrel but not rat PM. (**D** and **E**) Comparison of averaged relative force of contraction and relative resting tension (n = at least 3 in each experimental group) measured in the presence of CPA relative to control force of contraction before treatments. *, denotes statistically significant difference (P<0.05) defined by single group t-test comparing values with 1 in panel (**D**) and with 0 in panel (**E**). ‡, denotes significant difference (P<0.05) between relative resting tensions at 1.8 mM versus 3.6 mM of external Ca^2+^ in ground squirrel PM. (**F**) The profile of changes in the force of contraction and resting tension under manipulations with external Ca^2+^ and CPA was unaffected by the blocker of L-type Ca^2+^ channels, nifedipine, in line with moderate dependence of PM contractility on voltage-gated Ca^2+^ entry at low stimulation frequencies (**G**; *, P<0.05 for effects of nifedipine compared to corresponding controls; n = 4 in each group). (**H**) In ground squirrel PM, 2-APB reversed the contraction force potentiated by CPA to value below control. The averaged (n = 3) relative force of contraction is shown in panel (**D**).

The functional contribution of store-operated Ca^2+^ entry to regulation of PM contractility was assessed with the established protocol implementing a depletion/replenishment of intracellular Ca^2+^ stores [43,56,57] while testing the effect on Ca^2+^ influx of 2-aminoethyl diphenylborinate (2-APB), a widely-used nonselective regulator of store-operated channels [35,39,58]. Transition from 1.8 mM to 0 external Ca^2+^ levels, in the presence of CPA, gradually eliminated the contractility in both rat and ground squirrel PM (Fig 4A and 4B), in line with depletion of intracellular Ca^2+^ stores. Following reapplication of normal external Ca^2+^, while this CPA-induced prevention of SR Ca^2+^ replenishment strengthened the measured force of PM contraction in ground squirrels (Fig 4A and 4C), the lack of intracellular Ca^2+^ resources in rats led to only partial restoration of PM contractility (Fig 4B and 4D). Furthermore, in contrast to rat, ground squirrel PM exhibited a relatively brief but significant elevation of the resting tension (Fig 4A and 4B), indicating a transient Ca^2+^ overload presumably resulting from store-operated Ca^2+^ entry. Of note, such Ca^2+^ overload can be exaggerated by Ca^2+^ entry at doubled external Ca^2+^ levels (Fig 4C), which amplified the resting tension of PM to values comparable with the control force of contraction (Fig 4D and 4E). Outstandingly, PM in hibernating ground squirrels, despite the suppression of SERCA activity by CPA, were capable of withstanding a significant Ca^2+^ overload and enforcing increased contractility, emphasizing a remarkable plasticity of Ca^2+^ homeostatic mechanisms in cardiac muscle of hibernators (Fig 4A–4E).

Voltage-gated Ca^2+^ entry via L-type Ca^2+^ channels represents a major mechanism of external Ca^2+^ entry contributing to excitation-contraction coupling. However, suppression of L-type Ca^2+^ channels by nifedipine resulted neither in elimination of notches in resting tension nor in enhancement of the contractile force, following depletion of intracellular Ca^2+^ stores (Fig 4F and 4G). Nifedipine approximately halved the force of contraction in both rat and ground squirrel PM, indicating the contribution of this L-type Ca^2+^ channel-dependent Ca^2+^ entry at low stimulation rates (Fig 4G). According to the established contribution of different Ca^2+^ sources into excitation-contraction coupling in these animal species, this antagonist of L-type Ca^2+^ channels had a more profound effect on PM contractility at high stimulation frequencies (Fig 4G). It is noteworthy, that in the presence of nifedipine and absence of CPA, removal of external Ca^2+^ apparently was insufficient to significantly reduce abundant Ca^2+^ levels within SR of ground squirrels, since reapplication of external Ca^2+^ normally restored the contractile levels, but did not potentiate the force of contraction, which was seen in the presence of CPA (Fig 4F). Thus, the existence of alternative Ca^2+^ entry allowed suggesting the contribution of SOCE in regulation of PM contractility in ground squirrels, which was further confirmed by application of 2-APB that not only effectively reversed the CPA-induced potentiation of contractility, but also reduced the contractile force to values significantly lower than control (Fig 4C and 4G).

**Fig 5:**
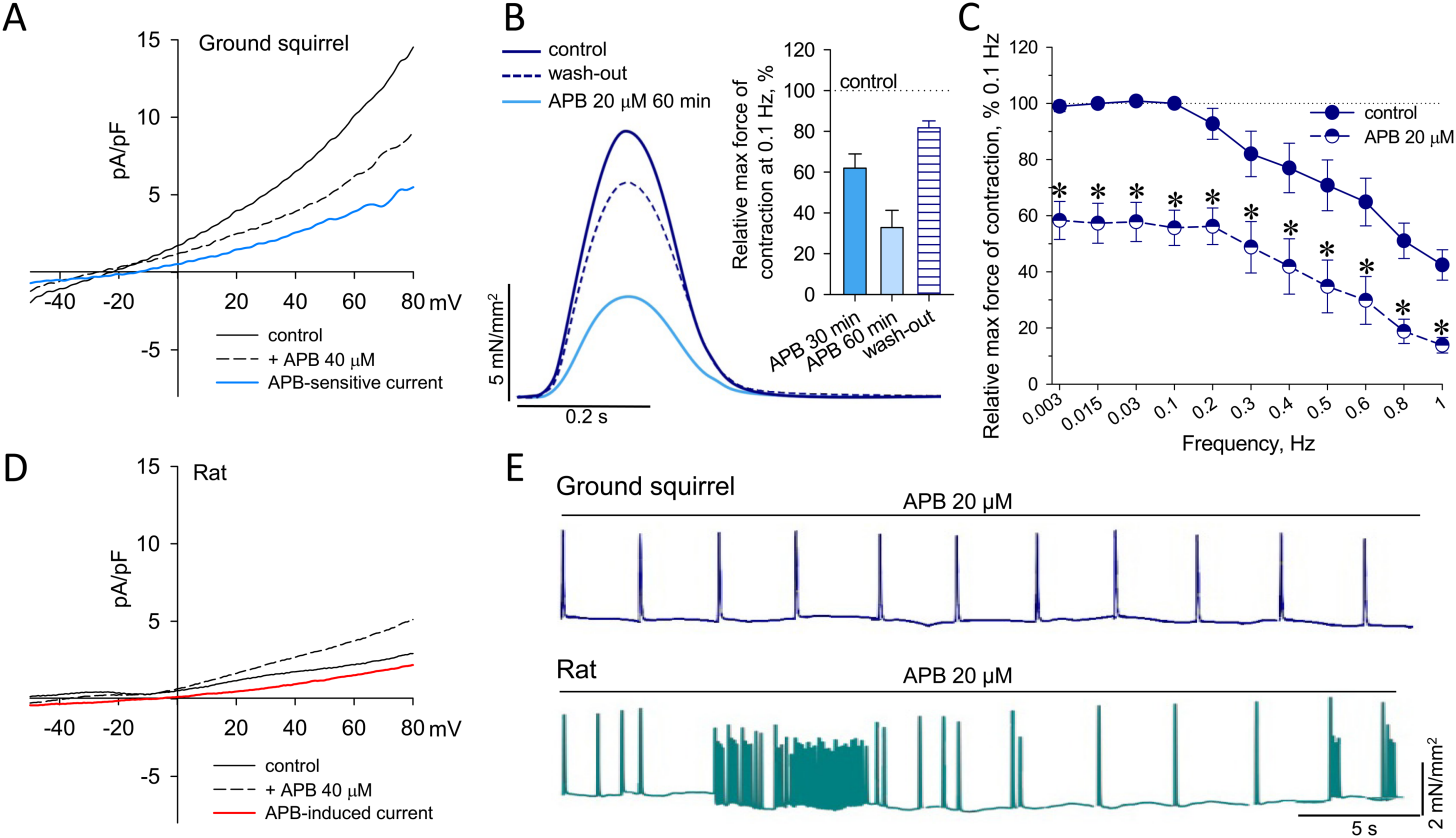
Effects of 2-APB on membrane currents in isolated cardiomyocytes and PM contractility in ground squirrel and rat hearts. (**A**) Representative voltage-current relationships of whole-cell membrane currents in a ground squirrel cardiomyocyte measured in response to a voltage ramp of 133.3 mV/s in the absence and presence of 2-APB. 2-APB-sensitive currents were obtained by subtraction of currents measured in the presence of 2-APB from control values at each membrane potential. (**B**) Time course and summary statistics of reversible 2-APB-induced inhibition of ground squirrel PM contractility measured at 0.1 Hz stimulation frequency. (**C**) FFR in ground squirrel PM in the absence (n = 8–9) and presence (n = 6) of 2-APB (20 μM); *, denotes statistically significant difference with P<0.05. (**D**) Representative voltage-current relationships of whole-cell membrane currents in a rat cardiomyocyte detected in response to a voltage ramp of 153.3 mV/s in the absence and presence of 2-APB. 2-APB-induced currents were obtained by subtraction of currents measured in the presence of 2-APB from control values at each membrane potential. (**E**) Representative recordings at 0.3 Hz stimulation frequency exemplifying the incidents of contractile dysfunction and arrhythmia induced by 2-APB in rat, but not in ground squirrel PM.

The impact of SOCE on Ca^2+^ homeostasis in hearts of hibernating ground squirrels was further probed in isolated cardiac myocytes. Under suppressed voltage-dependent K^+^, Na^+^ and Ca^2+^ currents, 2-APB (40 μM) inhibited whole-cell membrane current in ground squirrel cardiomyocytes (Fig 5A). Subtraction of the currents measured in the presence of 2-APB from control records revealed a non-selective 2-APB-sensitive current component with reversal potential slightly more negative than 0 mV (E_r_ = -11.7 ± 5.2 mV, n = 3), which is typical for non-selective SOCE measured in heterologous expression systems [59,60] or in adult cardiomyocytes [45,61]. However, in ground squirrel cardiomyocytes 2-APB-sensitive currents were detected without a pretreatment aimed at depleting intracellular Ca^2+^ stores, indicating that the detected current could be induced by certain Trpc-Orai1-Stim1 channels capable of operating in store-independent mode [36,40,62]. Concomitantly, in isolated PM of ground squirrels 2-APB reversibly suppressed the contraction force measured at 0.1 Hz of stimulation frequency, yet complete inhibition (32.7±8.5% of control values, n = 3) was reached approximately in one hour following drug application (Fig 5B). While this effect of 2-APB was detected throughout the stimulation range (Fig 5C), the most profound suppression of the contractile force was found at the lowest stimulation frequencies (below 0.3 Hz) where the contribution of voltage-sensitive Ca^2+^ entry to myocardial contractility is considered to be minimal [46,51,53]. SKF-96365, an alternative SOCE antagonist, also inhibited the contraction force of ground squirrel PM throughout the range of stimulation frequencies (S3 Fig).

In contrast to the effect in ground squirrels, 2-APB increased membrane currents in isolated rat cardiomyocytes (Fig 5D). 2-APB-induced current components (Fig 5D) exhibited reversal potential (E_r_ = -7.8 ± 4.2 mV, n = 3) similar to 2-APB-sensitive current in ground squirrels, yet with significantly reduced current density (0.39 ± 0.16 pA/pF, n = 3 vs. 1.52 ± 0.31 pA/pF, n = 3; P<0.05; at +20 mV membrane potential). In isolated PM from rats, Ca^2+^ entry provoked by 2-APB at concentrations exceeding 10 μM usually induced arrhythmia and sporadic contractile dysfunction, which was identified in all 5 performed experiments, whereas in PM from ground squirrels this agent reduced contractility without any proarrhythmic effects (n = 6; Fig 5E). Thus, these observations allow suggesting that ground squirrels, in contrast to non-hibernators, may reserve Trpc-Orai1-Stim1 channels to maintain cardiac contractile function during the transition between normal and hypothermic states.

### SOCE in control of PM contractility during cooling-reheating cycle

**Fig 6:**
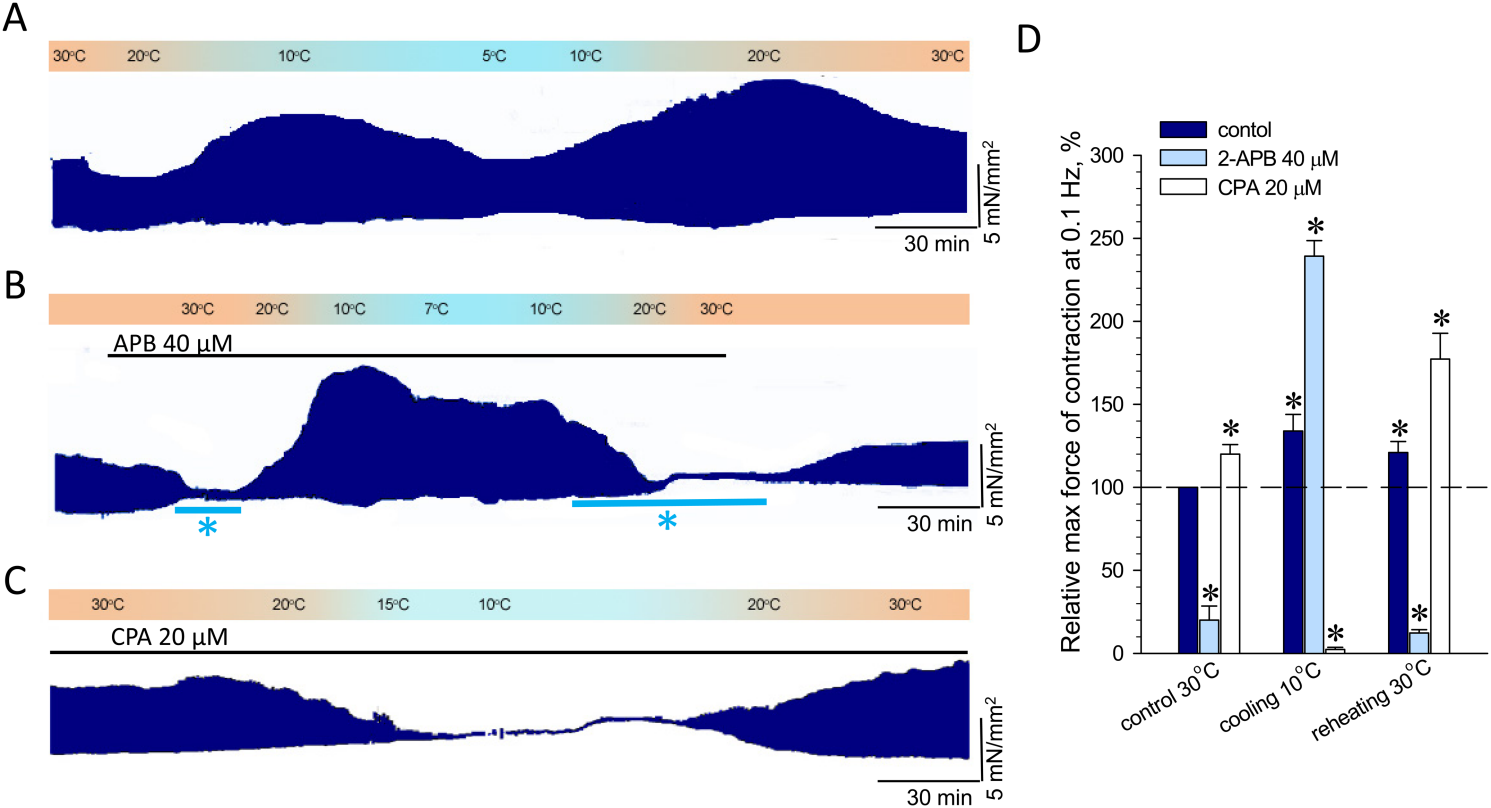
2-APB- and CPA-induced modulation of ground squirrel PM contractility during cooling-reheating cycles. PM contractility acquired at 0.1Hz stimulation frequency in control (**A**), in the presence of 2-APB (**B**) or CPA (**C**). The temperature of bath solutions was changed at the approximate rate 0.1–0.2°C/min. Blue solid bars marked by asterisks (**B**) indicate 2-APB-induced inhibition of PM contractility before and after cooling, respectively. The profiles of temperature changes and drug application are shown by corresponding colored gradient bars and solid lines above the traces. (**D**) Summarized statistics for (**A**), (**B**) and (**C**) at the indicated temperatures; *, denotes statistically significant differences (P < 0.05; n = 5, 4 and 3 in control, in the presence of 2-APB and CPA groups, respectively), estimated using the single group t-test compared to peak force of contraction before cooling (100%).

In order to assess the role of SOCE in ground squirrel myocardium during transition from high temperature to hypothermia, we compare contractility of PM in the presence of 2-APB during a slow cooling/reheating cycle at the rate of ~0.2°C/min from 30°C to 7–10°C and back to 30°C. The representative control recoding (Fig 6A) demonstrates that the operation capacity of PM during cooling-reheating cycle underwent significant changes in contractile force. The phase of attenuation in force of contraction at ~20°C gave place to the significant elevation of contractility during further cooling to ~10°C and to partial drop of peak contractile forces at the lowest temperature points. Reheating back to 30°C was characterized again by the increased contraction force at ~20°C followed by the restoration of initial contractile function, which remained somewhat elevated at approaching 30°C, compared to control contractility prior to cooling (Fig 6A and 6D). Surprisingly, the suppression of contractile function induced by 2-APB at 30°C was reversed and even further amplified following the cooling progression to ~10°C, despite the continuous presence of the SOC inhibitor. However, 2-APB effectively prohibited the restoration of PM contractile function following reheating to 20°C and 30°C, until complete washout of 2-APB (Fig 6B and 6D). At the low temperatures, where 2-APB lost the ability to suppress contractility of PM, the dominant role in governing cardiac muscle contraction pertained to SR Ca^2+^ stores controlled by CPA-sensitive SERCA activity (Fig 6C and 6D). Such profile of regulation of contractile function of PM from hibernating ground squirrels indicate that 2-APB-sensitive SOCE can play a pivotal role during entering to hypothermic adaptation (30–25°C) of cardiac contractility and govern the restoration of contractile function throughout the exit from hibernating state.

## Discussion

In the present study, we have identified that inhibition of the ability of SERCA to replenish SR Ca^2+^ stores oppositely affected contractility of cardiac PM in hibernating ground squirrels versus non-hibernating rats. While inhibition of SERCA in rat PM resulted in an expected reduction of contractile function, the contractility of ground squirrel PM, under such conditions, was paradoxically potentiated at low pacing rates, where the dominant role in mediating excitation-contraction coupling belongs to intracellular Ca^2+^ stores [46,51,53]. Although we hypothesized that voltage-insensitive passive Ca^2+^ influx would be a favorable mechanism contributing to excitation-contraction coupling under hypometabolic/hypothermic states of hibernators, the involvement of the Ca^2+^ store-associated functions in control of PM contractility was identified here only at the relatively high physiological temperatures during entry or exit from bouts of torpor. Thus, ground squirrels may adopt an unrecognized complementary mechanism supporting the reversibility of cardiac muscle contractility during transition of such heterothermic animals between their physiological states.

An established system that can be triggered by inhibition of SERCA due to depletion of SR Ca^2+^ stores is store-operated Ca^2+^ entry (SOCE) through the plasma membrane [41]. Stim1, an endoplasmic reticulum Ca^2+^ sensor, Orai1, a functional membrane unit of SOC, and the canonical Trp isoforms are putative constituents of cardiac SOC [35,36,38,45]. In particular, 6 isoforms of nonselective cation Trpc channels are grouped into 2 subfamilies based on structural and functional features: Trpc1/4/5 and Trpc3/6/7 [63]. It has been argued that Trpc3/6/7 can be activated via G protein-coupled receptor/phospholipase C signaling, whereas Trpc1/4/5 current can be induced by stretch or depletion of SR Ca^2+^ stores [40]. While members of the Trpc family along with Orai1 and Stim1 have been predominantly recognized in non-excitable cells, their role in regulation of Ca^2+^ homeostasis in cardyomyocytes has been associated with pathological cardiac remodeling [42,43]. In fact, Trpc channels are present at very low levels in normal adult cardiac myocytes, yet select Trpc isoforms have been found to be overexpressed in several animal models of myopathy, cardiac hypertrophy and heart failure [42,64–66]. Hence, the argument could be advanced that SOC represent an important element of cardiac adaptive mechanisms, which partially or completely has been lost in the tissue of non-hibernators, but remains preserved in hibernator’s hearts to support cardiac survival under environmental stress conditions.

Therefore, the expression of these SOC components was tested here in the papillary muscles of hibernating ground squirrels. We identified that the genes encoding all suggested SOC constituents were dramatically overexpressed in ground squirrel PM, compared to PM of non-hibernating rats. However, for instance, the relative 10^6^-fold increase in *Trpc7* mRNA expression identified by qPCR in ground squirrels compared to rats, may not necessarily indicate its redundancy in the myocardium of hibernators, but rather may be due to the very low levels of these transcripts in rat tissue. Surprisingly, the expression levels of particular mRNA and Trpc proteins, except Trpc3 protein, were not different in hibernating (winter interbout) versus summer active ground squirrels, suggesting that the mobilization of SOC function may rely on signaling mechanisms. This suggestion is supported by our observation that PM of summer ground squirrels although can functionally withstand experimental hypothermia, lack characteristic potentiation of contractility at low temperatures as well as CPA-induced elevation of contractile function (S1 and S2 Figs). One may speculate that critical Ca^2+^ store-related protein structures are constantly preserved in the cardiac muscles of ground squirrels, and can be operatively recruited during the transition to hibernating state. Indeed, studies have shown that in addition to regulation via G protein-coupled receptors, SOCE in cardiomyocytes is sensitive to changes in glucose homeostasis, hypoxic or ischemic events [67–69], characteristic of the transition of hibernators between active and torpid states [12,22,23].

Unfortunately, in the present study we cannot specify the exact composition of SOC in ground squirrel myocardium, which would require future extensive investigations implementing, for example, gene silencing techniques. However, in cardiomyocytes isolated from hearts of hibernating ground squirrels, testing using the whole-cell mode of the patch-clamp technique revealed the non-selective SOC activity without drug-induced depletion of intracellular Ca^2+^ stores [35,38,41]. According to present-day understanding, the Stim1-Orai1 complexes are responsible for plasma membrane conductance via Ca^2+^-release activated channels (*I*_CRAC_) that are highly selective to Ca^2+^ ions [35–37]. In contrast, Trpc channels do not contribute to *I*_CRAC_ [70] and in combination with Stim1 or Stim1-Orai1, produced less selective SOC membrane currents (*I*_SOC_) that are permissive to other cations [40,70,71]. Indeed, the reversal potential near 0 mV, detected for the 2-APB-sensitive membrane current in ground squirrel cardiomyocytes, indicates a non-selective *I*_SOC_, since *I*_CRAC_ would exhibit a reversal potential near the equilibrium potential for Ca^2+^, which under the experimental conditions used here was estimated within a range of +150 to +170 mV [32]. Despite the canonical definition of SOC as a unit sensitive to intracellular Ca^2+^ stores, such principle of their operation may not be applicable to all conditions in which Stim1 clustering results from the store depletion by inositol-1,4,5-trisphosphate-induced Ca^2+^ release or by inhibition of SERCA [62,72]. Cardiomyocytes are different from non-excitable cells in that they produce robust excitation-induced release of Ca^2+^ from intracellular stores that, under certain conditions, may reduce SR Ca^2+^ levels to the threshold of SOC activation. However, it is not evident that Ca^2+^ stores in ground squirrels PM are significantly depleted during excitation [46,73], as shown here using post rest potentiation measurements (Fig 2). Furthermore, 2-APB sensitive current components were measured in ground squirrel cardiomyocytes in the presence of nifedipine, which by blocking L-type Ca^2+^ channels would prevent RyR-mediated Ca^2+^ release from SR. Thus, we are tempted to suggest that in ground squirrel cardiomyoctes, the overexpression of Stim1 may provoke creation of punctae [38,41], which following association with abundant Trpc/Orai1 can lead to formation of SOC and activation of 2-APB-sensitive currents. Concomitantly, we identified that 2-APB-induced SOC inhibition resulted in significant reduction of contractile force measured in ground squirrels PM within the whole range of applied stimulation frequencies. Application of 2-APB to rat cardiomyocytes in our patch-clamp experiments resulted in activation of whole-cell membrane currents, emphasizing a striking difference between SOC regulation and, presumably, the channel composition in hibernating versus not-hibernating species [74]. Our observations are in accord with the previously reported ability of 2-APB to stimulate store-operated Ca^2+^ entry in some cells [56] and produce atrial and ventricular arrhythmias in rat and mouse hearts [75,76]. Indeed, it has been demonstrated that 2-APB can activate different types of Orai channels independently of Stim1 or Ca^2+^ store depletion [77]. This data suggest that Ca^2+^ entry via voltage-independent ion channels may elicit ectopic electrical activity leading to ventricular fibrillation and persistent or paroxysmal tachycardia [75,76]. Thus, while upregulation of SOCE in non-hibernator hearts suggested to be deleterious [35,43,63,64], under pathological remodeling it may be viewed as an endeavor to mobilize the obsolescent Ca^2+^ controlling mechanism that is still in use by hibernators.

Since the physiological significance of SOCE remains elusive, especially in excitable cells, such as cardiomyocytes, we can only outline their possible contribution to Ca^2+^ homeostasis and myocardial contractility in hibernating ground squirrels. Permissive for Ca^2+^ ions, SOC can serve as a scaffold for local signaling complexes that directly sense the proximal Ca^2+^ emerging from the channel [63,78,79]. It has been suggested that in such capacity SOC can provide local Ca^2+^ in specific microdomains to reload particular SR compartments that are distinct from the greater SR involved in regulating contractility [63]. On the other hand, non-selectivity of Trpc channels, with a Na^+^/Ca^2+^ permeability ratio (P_Ca_/P_Na_) between 1 and 1/10 [80,81], at physiological concentrations would allow significant Na^+^ load in cardiomyocytes. This mechanism can contribute to the spatial Na^+^ accumulation, which can be sufficient to drive Ca^2+^ entry via reverse mode of NCX [82,83], suggested for hibernators [6,46,84]. Finally, it has been demonstrated that, by binding phospholamban, an endogenous SERCA inhibitor, Stim1 can facilitate SERCA-dependent refiling of SR by Ca^2+^ [62], which in turn would result in a positive inotropic effect.

Despite the absence of a clear understanding of the proposed mechanisms for SOC-dependent control of contractile function in hibernating ground squirrel PM, direct testing of SOC inhibition during a cooling-reheating cycle revealed the clear contribution of 2-APB-sensitive components to hypothermic adaptation of cardiac contractility. We conclude that, in contrast to non-hibernating species, hibernating animals evolutionary retain the SOCE-dependent mechanism to secure adaptation of cardiac contractility during transitions in and out of hibernating states. Further investigation of this phenomenon may open a new pathway to comprehension of the protective mechanism governing cold tolerance in the myocardium of hibernating animals.

## Supporting information

S1 Fig Typical contractile profile of PM from summer ground squirrel during cooling-reheating protocol. (PDF)

S2 Fig CPA did not affect force-frequency relationships of PM in summer ground squirrels. (PDF)

S3 Fig SKF-96365 supressed contractility of PM isolated from ground squirrel hearts. (PDF)
