# Supplementary material for: Store-operated Ca2+ entry supports contractile function in hearts of hibernators
Source: PLoS One. 2017 May 22;12(5):e0177469. doi: 10.1371/journal.pone.0177469 (PMC5439705; doi:10.1371/journal.pone.0177469)
Supplement: S1 Fig — (PDF) [file pone.0177469.s001.pdf]

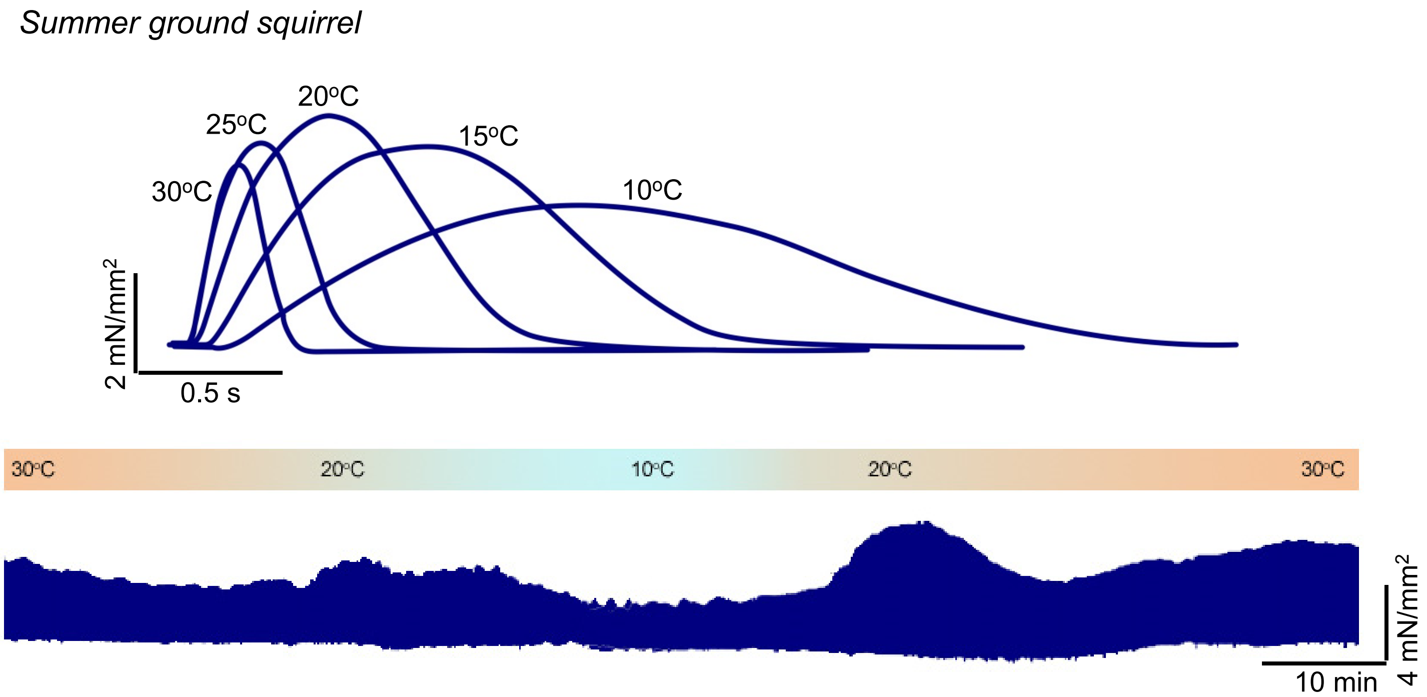

Representative recordings of the isometric contractions of papillary muscle (PM) isolated from the heart of summer ground squirrel (GS) and corresponding changes in PM contractility during cooling-reheating protocol (stimulation frequency 0.1Hz). The profiles of temperature changes is shown by colored gradient bars above the trace. In contrast to winter interbout GS, PM of summer GS exhibited a minor potentiation of the contractile force at low temperatures, yet, in contrast to rats, fully restored contractile function at the end of reheating back to 30°C.
