# Supplementary material for: Store-operated Ca2+ entry supports contractile function in hearts of hibernators
Source: PLoS One. 2017 May 22;12(5):e0177469. doi: 10.1371/journal.pone.0177469 (PMC5439705; doi:10.1371/journal.pone.0177469)
Supplement: S2 Fig — (PDF) [file pone.0177469.s002.pdf]

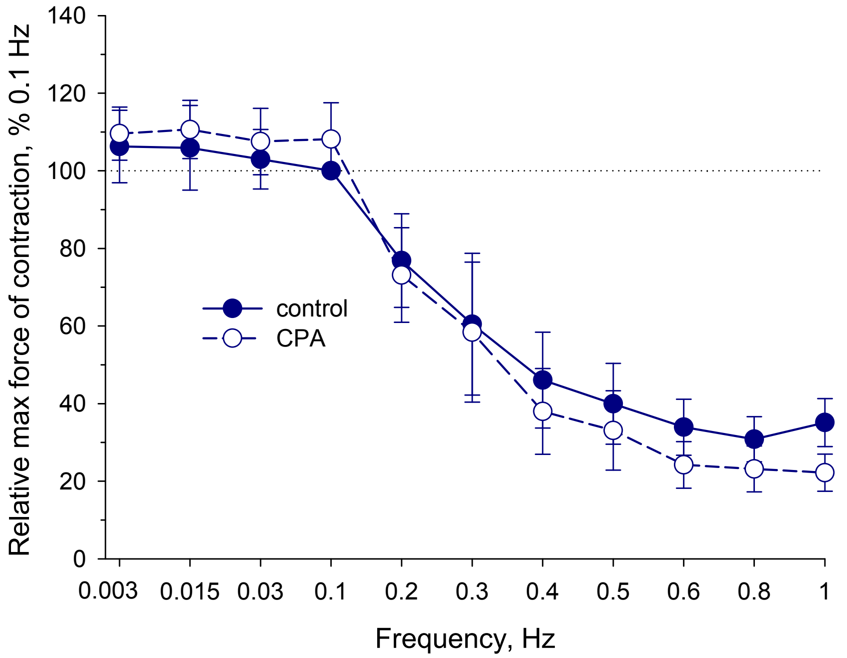

Force-Frequency-Relationship (FFR) in papillary muscles of summer ground squirrels (n = 3) in the absence and presence of 10 μM CPA were constructed as described in Figs 1C and 1D. Effect of CPA was insignificant throughout the range of stimulation frequencies.
