# Supplementary material for: Store-operated Ca2+ entry supports contractile function in hearts of hibernators
Source: PLoS One. 2017 May 22;12(5):e0177469. doi: 10.1371/journal.pone.0177469 (PMC5439705; doi:10.1371/journal.pone.0177469)
Supplement: S3 Fig — (PDF) [file pone.0177469.s003.pdf]

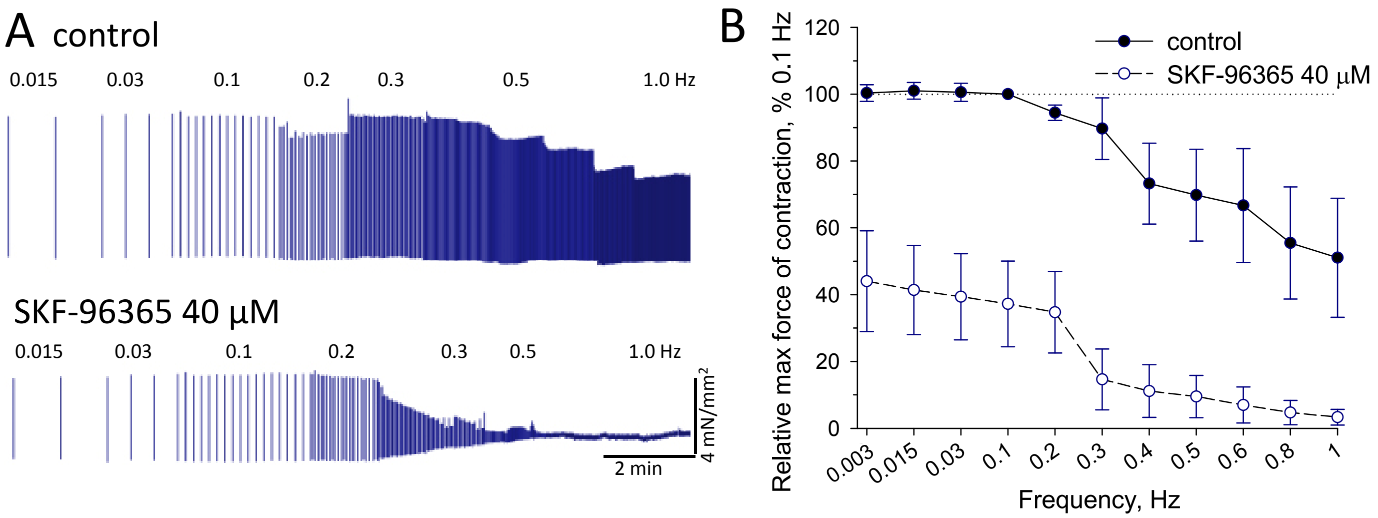

The effect of SKF-96365 on the isometric force of papillary muscle (PM) contraction (**A**) and corresponding Force-Frequency-Relationships (FFR) (**B**). SKF-96365 significantly suppressed PM contractility in hibernating interbout ground squirrels (n = 3) throughout the range of stimulation frequencies. The values for FFR were calculated relative to the control force of contraction at 0.1 Hz.
